# Key Performance Indicators Applied in Medicine

**DOI:** 10.1055/s-0045-1809426

**Published:** 2025-09-26

**Authors:** Ricardo Ferreira Bento, Mara Edwirges Rocha Gândara, Silvio Pires Penteado

**Affiliations:** 1 Department of Otorhinolaryngology, Faculty of Medicine, Universidade de São Paulo, São Paulo, SP, Brazil

**Keywords:** health care quality indicators, institutional management teams, quality improvements, health services research, knowledge discovery

## Abstract

**Introduction:**

Key performance indicators (KPIs) measure the critical functions and activities that must be performed in an organization to provide management with an assessment of quantity and quality, from which it can aid strategic decisions to achieve objectives.

**Objective:**

To map the literature review on KPIs in the context of medicine.

**Methods:**

In the literature review, we adopted a proposed model and made appropriate adaptations to it. The PubMed database was chosen, with filters applied to define a path. The findings of the articles were briefly discussed in comparison with others to highlight specific findings.

**Results:**

Almost 100 research articles were eligible for investigation in a wide range of third- and second-degree medical specialties. In these articles, the KPIs are numbered from 1 up to 797 qualitative and quantitative measures.

**Conclusions:**

The KPIs are not limited to a financial regime; instead, in the field of medicine, they play an important role in a wide range of medical specialties, from clinics, therapeutics, surgery, pharmacy, research, regulatory affairs, hospitalization, management, and product development. These indicators can be implemented and evaluated by different means, from simple resources such as home visit, telephone survey, cross-comparison with a benchmark, questionnaires, spreadsheets, to the most sophisticated technology, such as IT resources, artificial intelligence, the linear hinges model, the Research and Development/University of California, Los Angeles (RAND/UCLA) appropriateness method, and the Monte Carlo simulation.

## Introduction

In a systematic review on measurements and improvements during the coronavirus disease 2019 (COVID-19) pandemic, Amer et al. 1 categorized 797 key performance indicators (KPIs) that resulted in 45 subdimensions: finances, efficiency and effectiveness, availability and quality of manpower and supplies, development of health professionals, evaluation of managerial tasks, responsibilities of health professionals, time metrics, safety and elimination of risk factors, communication, community and reputation, and technological resources.

At a Cancer Detection Centre in Belgium, a 15-year extension of the population-based mammography screening program found that most of the KPIs met the European Union (EU) benchmarks, despite the suboptimal attendance rate. These authors stated that KPIs “cannot replace a mortality analysis, but enable programs to compare performance against objectives”. 2 These indicators are specifically designed and measurable metrics of being used within healthcare to measure performance by acting as ‘flags’ or ‘alerts’ to identify good practice, provide comparability, as well as to identify areas for improvement within a service allocation. 3 This concept was used in one Brazilian study meaning “a quantitative measure that can be used to track an organization's progress and allows the monitoring, decision-making, and quality improvement”. 4

Emergency medicine has proven that a survival benefit was identified when the response time was up to four minutes for patients at intermediate or high risk of mortality. 5 The adoption of a health service performance framework as a platform for a set of specific, measurable and action-oriented KPIs would be a positive step forward. 6 Simple but important qualitative measurements at emergency care demonstrate that “there is a need for less simplistic quality indicators which recognize that there are many stages between a patient's call for help and safe arrival in hospital”. 7 .

In time, KPIs were implemented in several medical specialties, including Anesthesiology, 8 Cardiology, 9 10 11 Coloproctology, 3 12 Community Health Sciences, 13 Dentristy, 14 eHealth, 15 Emergency, 16 17 18 Endoscopy, 19 Endocrinology, 20 21 Epidemilogy and Infection, 22 Genetics, 23 Hospital Management, 24 Intensive Care, 25 Medical Education, 26 Mental Health, 27 Nephrology, 28 Nursery, 29 Nursing and Midwifery, 30 31 Oncology, 32 Orthopedic Surgeon, 33 Optopmetry, 34 Otolaryngology, 35 36 Palliative Care, 37 Phatology, 38 Pharmacy, 4 39 Plastic Surgery, 40 Prosthetic and Orthotic, 41 Primary Healthcare, 42 Psychiatry, 43 Radiology, 44 45 46 Reprodution, 47 48 49 Speech Pathology, 50 Sports Medicine, 51 52 53 Urogynecologist, 54 and Therapeutics Innovation. 55

Our team has identified tangible KPIs that are not limited to the articles cited in the foreground, but which provide a review of the literature in medicine with restrictions on time, database and type of research, as we will describe later.

Hence, our objectives are to collect a set of articles in medicine in line with KPIs; to carry out a descriptive analysis of the literature on the subject, where applicable; and to summarize elements of its main conclusions, where applicable.

## Methods

**Fig. 1:**
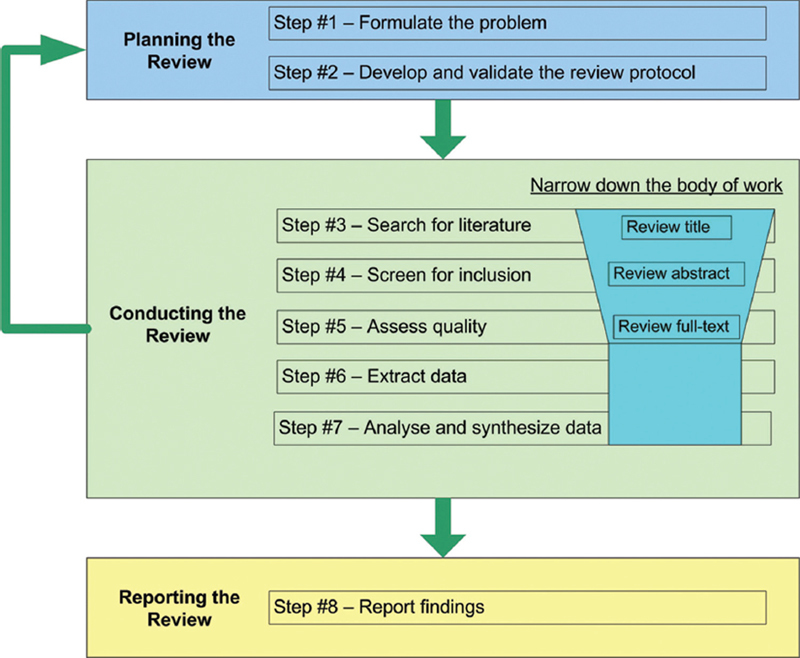
Process of literature review. **Note:** Adapted from Xiao and Watson. 56

The present article is organized based on a template designed by Xiao and Watson 56 for literature reviews ( Fig. 1 )

### Step 1–Formulate the Problem

Instead of zooming out too many research questions, we follow the footprints to formulate a specific problem and then look at how to map and solve that question. Cronin et al. 57 suggested a theoretical framework to answer three questions: “Has a conceptual or theoretical framework been identified? Is the framework adequately described? Is the framework appropriate?” Which is what we follow here.

### Step 2–Develop and Validate the Review Protocol

In short, it is a predefined plan that specifies the methods for carrying out the review. The review protocol increases the reliability of the research, as readers can use the same protocol to repeat the study for cross-checking and verification.

Our initial research found 7,720 articles. At the second try they were reduced to 1,219 articles, but both cases could be labeled as a large sample for the literature review. Our review protocol needed to move forward for the sake of reasoning.

### Step 3–Search for Literature

We value access to full texts more than abstracts; that is why we used the virtual private network (VPN) of Universidade de São Paulo (USP) to search PubMed to ensure a secure connection and access the full texts.

Refining our research led us to filter article type as a set of combinations: Action Research, Cohort, Cross-Sectional, Design of Experiments, Explanatory Design, Exploratory, Gap Analysis, Observational, Narrative Review, Randomized Controlled, Retro-Prospective, Retrospective Comparative, Phenomenological Methodology, Prospective, Prospective Cohort, Longitudinal, and Study Protocol.

We used the filters as defined: Human, English, Male and Female, and, lastly, only MEDLINE. The timeframe was set as 17 September 2019 to 17 September 2023, which can be justified by considering recent research as more relevant to the current situation, therefore being able to provide more useful insights. 56 There were 58 positive results for our criteria at this point. Furthermore, MEDLINE provided tools such as the possibility of sending the search results by e-mail, and just a .txt file with abstracts and a .cvs file with the main components of the search.

### Step 4–Screen for Inclusion

Once more, Xiao and Watson 56 paved the way through an efficient two-step procedure: first, a general search of articles for inclusion based on a review of the abstracts, followed by a refined quality assessment based on a full-text review. As our literature search did not cover a large proportion of the medical specialties we originally sought, we were obliged to add another 51 articles.

We noticed that some summaries were blurry, so we had to search in the text itself, for example, when the term Quality is recorded 58 59 59 60 or when a Biomaterial is set. 61 Some researches are still in progress; 62 63 64 however, they were still considered for our literature review.

### Step 5–Access Quality

In time, two texts were excluded: Halling et al., 65 as it was not focused on health care or KPIs; and Nicholson's study, 66 which is conceptual. Thus, the final results led to 56 articles used as primary data. Complementary literature was also used to support our work.

### Step 6–Extract Data

Our cache of 58 articles ( Supplementary Appendix 1 ; online only) from PubMed were organized in a spreadsheet-like format where we preserve authors ‘original own words rather than put our own version to maintain authors’ concepts. On the column Main Findings, you expect to have full authors' findings when that sentence ends with period. In case authors' findings cannot fit completely, we adopt parentheses with ellipsis points inside. We invite the readers to access the full text for a broader comprehension. The authors' text was totally preserved. “Copy and paste” was our data extraction pattern to preserve the authors' fundamentals.

### Step 7–Analyze and Synthesize Data

Sandelowski et al. 67 highlights the problems faced when combining qualitative (such as differences in ontological positions, epistemological positions, research paradigms, fundamental theories, philosophies, and methodologies) with quantitative literature (such as the heterogeneity of the studies). Both methods are important, as our study did not aim to cross-compare the articles or to determine which of them is more relevant on the topic of KPIs. Therefore, we omit our opinion.

### Step 8–Report Findings

**Fig. 2:**
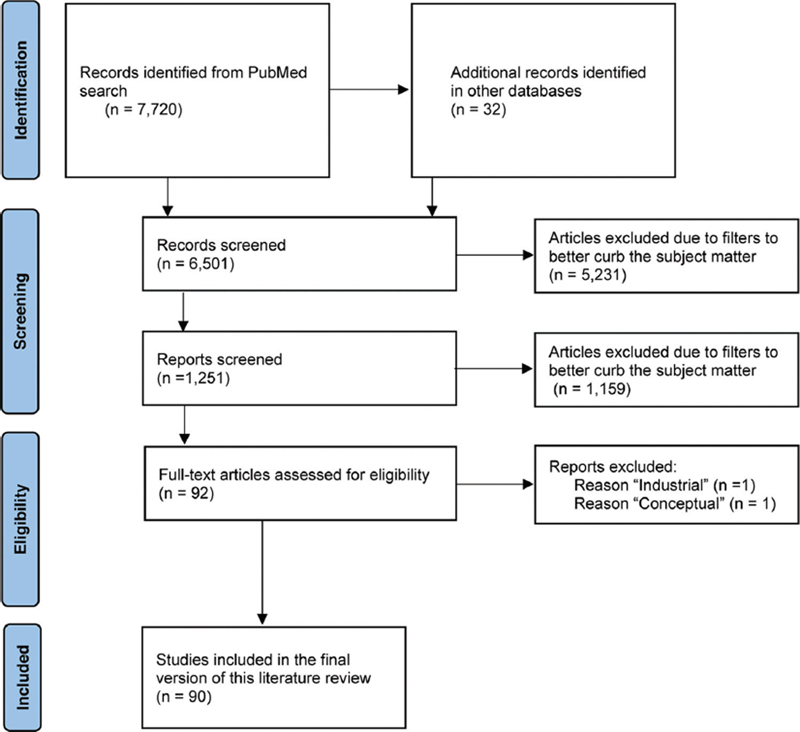
Screening flow of the present article, following the Preferred Reporting Items for Systematic Reviews and Meta-Analyzes (PRISMA) statement.

Our primary choice treasures the convenience and clearness of spreadsheet-like format with Article, Declared Area of Interest / Related Sciences, Type of Experiment/Study, KPIs Method, KPIs Quantity, and Main Findings / Results. Our search and filtering flow are shown in Fig. 2 .

## Results

### Descriptive Analyses

**Fig. 3:**
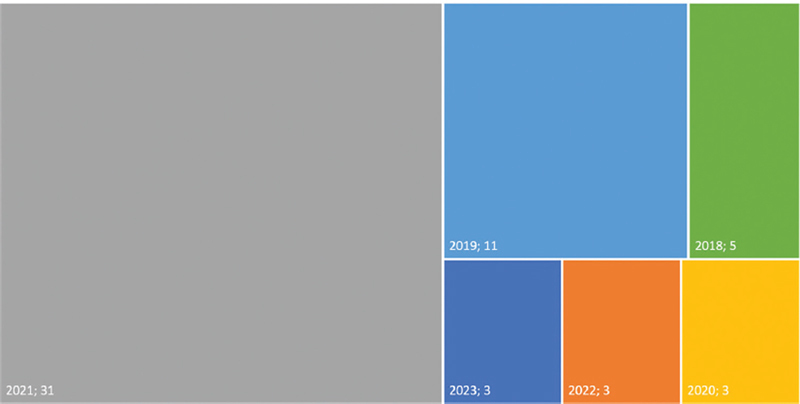
Literature evolution.

Our temporal analysis tells us 2021 was the most prolific year with 31 publications (55%), followed by 2019 with 11 publications (20%), then 2018 with 5 publications (9%). The lowest ratios were found in 2023, 2022, and 2020, with three publications each (5% each), as shown in Fig. 3 .

We don't have the space to discuss each of the final 56 articles, but some deserve a few observations in the light of the KPIs.

### Selected Articles (2018)

Two articles have a cross-comparison results with Government or Economic Bloc guidelines: Fitzpatrick et al. 68 and Philippon et al. 69 The former covers the national BreastCheck screening program in the Republic of Ireland over a period of more than a decade, compared with the standards of the program and European guidelines. Authors highlight that the “Adoption of a longitudinal perspective on screening programs and examination of their performance indicators over a sustained period is worthwhile” on anticipating a trend: The uptake of BreastCheck examinations remained above the acceptable standard of 70% throughout the period but has been falling since 2010.

The latter article makes comparative quantitative analyses of the Canadian and Australian health systems to affirm the comparatively superior performance of the Australian system on most indicators, concluding that Canada needs to adopt a more serious approach to the federal role and the introduction of a national pharmaceutical program. In one of them, 69 the authors sent a message to authorities: “It is hoped that this article will empower health leaders to take action in these areas.”

### Selected Articles (2019)

Jones et al. 70 developed a mortality and discharge modeling of hospitalized stroke patients using a Markov-based phase recovery (stochastic model used to model systems that change pseudo-randomly) for patients with transient ischemic attack (TIA), noting that “the likelihoods of death and discharge to nursing home increase with age, and the likelihood of discharge to usual residence decreases with age.”

In the Netherlands a published by Dohmen and van Raaij 71 defined 10 KPIs for cataract surgery without flags, just plain values for perioperative complications (maximum: 1%), postoperative complications (maximum: 1.2%), conducted front vitrectomy (maximum: 0.2%), corrected visual acuity (≥ 0,5–96%), patient satisfaction of outpatient visit (≥ 90%), average grade by patients scale from 0 to 10 (≥ 8), net promotor score (≥ 55), availability of operating room (100%), lead time first hospital visit to operation (maximum: 30 days), and patient satisfaction with waiting time (≥ 9 0%).

Prospective research 72 on provisional colonoscopy certification (the transition from training to the new independent practitioner) stated that some KPIs exceeded the national standard of 90%. Trainees with declining performance incurred higher rates of moderate to severe discomfort after provisional colonoscopy certification, despite needing higher dosages of analgesics, and were more likely to need assistance from an instructor in failed procedures. A total of 257,800 colonoscopies were performed over a 6-year period.

### Selected Articles (2020)

An observational cohort study in Spain 73 had hypothesized that, due to the lower response to surgical aggression and lower rates of postoperative complications, enhanced recovery protocols after surgery may reduce colorectal cancer-related mortality. However, no updates were found for this research.

Across 16 competitive matches a total of 15 males outfield players (5 defenders, 6 midfielders, and 4 strikers), aged 16 to 18 years, from the club's scholarship squad were subjects to perform fitness testing battery under specific circumstances. Wing et al. 74 were intrigued about the importance of strength and power among these athletes. Results showed that there was a significant correlation between countermovement jump, squat jump, and total score of athleticisms in relation to heading success. In this case, the KPIs were passing, shooting, dribbling, tackling, and heading.

### Selected Articles (2021)

Ratnovsky et al. 75 aimed to perform an exploratory dig in data analysis to develop an admission prediction model based on a dataset that was constructed from KPIs selected by a panel of hospital administrators, physicians and nurses. The patients per day indicator was significantly higher on Sundays compared with all other weekdays ( *p* < 0.001) as it did not differ significantly between midweek days. The distribution on Fridays was significantly different from the rest of the weekdays and Saturday. The length of stay of patients' indicator did not differ significantly between Sunday and weekdays ( *p* = 0.288).

The British Orthopedic Association and British Geriatric Society published, in 2007, their care guide for patients with fragility fractures (Blue Book) in which the KPIs for hip fracture care were determined. Crozier-Shaw et al. 76 were drawn to analyze Blue Book compliance during the COVID-19 pandemic and analyze hip fracture outcomes, including the COVID-19 infection rate. They write of a 20% reduction in hip fractures during 2020, compared with 2019, but the pandemic has increased mortality associated with hip fractures, although not in a statistically significant way. They comment on a Chinese experience, particularly of a Wuhan study with 10 hip fractures and COVID-19, which showed a mortality rate of 40%.

One case of biomaterial development was listed on our sample. Photosensitizer nanoparticles emission for precise photodynamic oral cancer therapy as an alternative for drugs. Wu et al. 61 declared their 532 nm clinical laser irradiation could generate 3.78-fold more ^1^ O2 than clinically approved hemoporfin. Based solely on the mouse model of oral cancer, it was possible to demonstrate that AIEPS5-NPs-NB (material development and related technique) can lead to more than 90% tumor ablation under laser irradiation, and the inhibition rate was much higher than that of hemoporfin.

A tertiary hospital with a capacity of 1,300 beds, 1,654 items in the pharmacy, and 6 robotics systems that can serve 1 thousand patients daily have a related article due to the unpredictable COVID-19 experience. The development of a near real-time pharmacy dashboard can be useful in managing this workload, but it brought obstacles regarding the integration of different database platforms, such as the Oracle Database Server (Oracle Corp.) with SQL Database (Microsoft Corp.), in which the Cloverleaf engine (Infor) was chosen. All IT must comply with the Health Insurance Portability and Accountability Act at all stages of data extraction, transfer, manipulation and aggregation. Al-Jazairi et al. 77 point out that “minimizing therapy interruptions and improving the patient experience—all of which are becoming priorities for large-scale, complex organizations.” Here, we present a collection of 31 KPIs considered.

There were two articles highlighting KPIs in sports to measure athlete performance: one is about swimming 78 and the other regarding soccer. 79 The former measured lifesaving-specific skills (block time with fins, 50m swimming with fins, 25m mannequin carry, and 50m mannequin tow with fins), swimming skills (block time freestyle, 50m freestyle, 15m split time, 25m split time, tumble turn, 200m freestyle, and pace at 4 mmol/L), muscular strength and endurance (ventral core, lateral core, dorsal core, bench press, bench pull, and standing long jump), flexibility (shoulder inside rotation, shoulder outside rotation, hip extension, sit-and-reach, plantar flexion, and supination), and anthropometry data (age, height, arm span, and body mass), with a total of 27 KPIs. 78 The latter took a sample of 145 players from 42 countries, with 64.3% of practitioners using data tools and applications weekly, according to the KPIs. In a general run, 83% of participants reported the use of event data with technology. As 35% of participants reported using their own tagging within platforms, such as Hudl or Sportscode, rather than relying on external sources such as Stats Perform or official league sources such as Bundesliga Event Data. The most used KPIs were shots on goal (77%), shots from penalty area (73%), total shots (70%), crosses (70%), shooting efficiency (68%), shots from the goal box (57%), expected goals (46%), pass evaluation metrics (44%), space control (33%) and expected possession value (22%). 79

### Selected Articles (2022)

Colorectal surgery is still associated with a significant burden of postoperative complications and, ultimately, costs for healthcare. Massa et al. 63 launched an audit to evaluate its impact in a large area of northern Italy. All consecutive patients undergoing surgery for primary colorectal cancer between 15 April 2019 and 31 December 2023 were enrolled. This research is still in progress.

A study protocol by Opgenorth et al. 62 is still underway. Its aim is to better manage acute renal replacement therapy (KRT), which is administered to acutely ill patients to support kidney function and life in the Intensive Care Unit (ICU). Implementing standardized acute KRT pathways can ensure its safe and effective management. The Dialyzing Wisely Critical Care Strategic Clinical Network is a registry-integrated, interrupted time-series evaluation of the implementation of a standardized, stakeholder-informed, evidence-based acute KRT pathway. Dialyzing Wisely will implement, monitor, and report on a set of KPIs, along with a care pathway that will transform the quality of acute KRT in Alberta's ICUs as, at present, there is no standardized approach.

### Selected Articles (2023)

In Uganda almost 75% of the entire population is rural, while many healthcare workers are concentrated in urban areas. Minimizing such issues drove them to create community health workers (CHW) programs, with a team of 5 to 6 people as village health teams (VHTs). Agarwal et al. 80 argues that these programs face significant disputes, due to poor retention and insufficient financial investments. Furthermore, they mention threats to personal health, such as the COVID-19 and Ebola pandemics. This study is in progress and is KPIs-related.

The Shanghai Stroke Service System (4S) is a regional stroke network making use of an online database that automatically extracts data from structured electronic medical records. Stroke is one of the leading causes of death and disability in China. Patients who are 18-years or older with International Classification of Diseases 10th Revision (ICD-10) stroke codes (I63, I61, and G45) as the primary diagnosis at discharge were included in the study by Xu et al., with 11 KPIs listed. 58 All stroke and transient ischemic attack (TIA) admissions from participating centers, between January 2015 and December 2020, were included. Sample fetch consisted of 92,395 stroke or TIA admissions from 61 centers. The study concluded that improvement of stroke care can be better achieved by a regional management intervention basis, instead of a centralized one.

## Discussion

In the context of educational proceedings, the performance indicators can be traced back to 1880, as noted by Hogg (apud Fitz-Gibbon 81 ): “In the late nineteenth century both in Europe and the United States the growth of new methods of scientific measurement, for example the pioneering work of Edgeworth at Oxford in the 1880s, aided the development of tests to assess schoolchildren”.

Articles in the medical field that explore performance indicators date back to 1923. Robinson 82 stated that “to realize the main distinctions between different types of work, it is also important to realize the factors common to all types of human performance”. Later, in 1931, Symonds 83 expressed that measurements are needed to “include it only the measurement of intelligence and achievement, but also the non-intellectual qualities and characteristics variously know as character, conduct, traits, personality, emotionality (...).”

Along with these historical records, the present article makes relevant contributions to the field of medicine by revealing that KPIs are a constant in the practice of medicine. For example, this investigation presents studies with results over 35 medicine specialties ranging from clinical, 9 12 28 42 therapeutics or medication, 10 63 84 surgery, 8 24 31 36 laboratory and pharmacy, 4 39 77 novelty, 23 30 47 48 regulation, guidelines, and national protocols, 3 58 68 85 86 87 hospitalization, 11 72 88 exploratory, 13 14 15 16 17 18 19 20 21 22 25 27 29 32 80 management, 41 43 information technology, 44 89 90 and equipments 45 61 and sports. 51 52 53 74 78 79

A second contribution involves carrying out descriptive and thematic analyses together. The descriptive analysis provides a broader understanding of how medical researchers and academics have approached KPIs over time. The evolution of this subject through different methods and applications, in strictly controlled scenarios or in the uncertainty caused by the COVID-19 pandemic, may intrigue discerning readers to develop KPIs, both in medical practice and in the field of research. An in-depth investigation into how to overcome regular and routine obstacles is present here, for regulatory affairs or even for managers and engineers in related fields.

Another contribution of the presented article tells us the KPIs can be implemented and be evaluated by different means, such as household visit, phone survey, cross-comparison with a benchmark, questionary, IT, artificial intelligence, observational, linear hinges model, Research and Development (RAND)/University of California, Los Angeles (UCLA) appropriateness method, and the Monte Carlo simulation.

Private medicine has investors behind a decision-making process in a continuous flow. Return on investments, growth, profit margin, revenue, customer loyalty, inflation, taxes, regulation, competitors maneuvers, technology, mergers, acquisitions, and bankruptcies are all part of the intrinsic vocabulary, like non-medical, related fields (such as consumers goods, capital goods, services).

Regarding the public health sector, where almost none of this vocabulary is used, the Brazilian Unified Health System (Sistema Único de Saúde, SUS, in Portuguese) can be used as an example, as the country considers health a constitutional right. In a nutshell, SUS is universal and free for all in the fifth largest country in the world. Is there room to study and apply KPIs in a public health system like the SUS? Apart from monetary issues, does it make sense to increase efficiency and effectiveness? Is it possible to improve the availability and quality of supplies, as well as the development, training, and performance of health professionals, managerial tasks, safety, communication, reputation, and patient satisfaction in SUS?

Only two articles 35 36 on otorhinolaryngology, were found, which suggests that KPIs are relatively new to this field. With time, this method will improve diagnosis, prognosis, management, and therapy. Furthermore, artificial intelligence and machine learning can also apply KPIs with the aim of saving lives and time.

Lastly our research team does not propose a method, a guideline, or a model to implement KPIs as a general tool, which could be handy. This could be done in a future study, aiming to humanize KPIs, as it is currently only known as a financial tool.

## Limitations to the Study

A superficial view of this subject may lead the reader to look at opportunities to apply for KPIs in their professional routine. With this, the authors emphasize the need for more articles to be discussed with more latitude, more topics, with different points of view. The present could be called a *systematic review* .

## Conclusion

This literature review concerns the use of KPIs in the context of medicine, as analyzed in the areas of clinical practice, therapeutics, surgery, pharmacy, research, regulatory affairs, hospitalization, management, and product development. There is a symmetrical conflict between infinite demand and limited resources, which KPIs can play an important role in bridging, regardless of medical science, wealth or other barriers, as there are many resources to balance the challenge, such as information technology, artificial intelligence, the linear hinges model, the RAND/UCLA appropriateness method, and the Monte Carlo simulation.
