# Supplementary material for: Key Performance Indicators Applied in Medicine
Source: Int Arch Otorhinolaryngol. 2025 Sep 26;29(3):1–10. doi: 10.1055/s-0045-1809426 (PMC12473520; doi:10.1055/s-0045-1809426)
Supplement: Supplementary file 1 — Supplementary Material [file 10-1055-s-0045-1809426-s251927.pdf]

Supplementary Appendix 1 List of fifthy-eight articles with main findings

Article	Declared Area of Interest / Science Related	Type of Experiment/ Study	KPIs Method	KPIs Quantity	Main Findings/ Results
Agarwal S, Tweheyo R, Pandya S, Obuya E, Kiyomoto A, Mitra P, et al. Impact of a recognition package as an incentive to strengthen the motivation, performance, and retention of village health teams in Uganda: a study protocol for a cluster randomized controlled trial. Trials. 2023 Jun 23;24(1):428.	Community Health	Randomized Controlled	- Household visit<br>- Phone survey<br>- Report	3	Not defined
Xu J, Xie Y, Fang K, Wang X, Chen S, Liu X, et al. Effect of the Shanghai Stroke Service System (4S) on the quality of stroke care and outcomes: A prospective quality improvement project. Int J Stroke. 2023 Jun;18(5):599–606.	Stroke	Cohort	- Cross-comparison with a benchmark	11	The 4S intervention was associated with increased adherence to the stroke care guidelines, which further translated to improved clinical outcomes.
Opgenorth D, Bagshaw SM, Lau V, Graham MM, Fraser N, Klarenbach S, et al. A study protocol for improving the delivery of acute kidney replacement therapy (KRT) to critically ill patients in Alberta - DIALYZING WISELY. BMC Nephrol. 2022 Nov 16;23(1):369.	Nephrology	Study Protocol	- Cross-comparison with a benchmark	15	Dialyzing Wisely will implement, monitor, and report a suite of KPIs of acute KRT, coupled with a care pathway that will transform the(...)
Massa I, Ghignone F, Ugolini G, Ercolani G, Montroni I, Capelli P, et al. Emilia-Romagna Surgical Colorectal Cancer Audit (ESCA): a value-based healthcare retro-prospective study to measure and improve the quality of surgical care in colorectal cancer. Int J Colorectal Dis. 2022 Jul;37(7):1727–38.	Colorectal Disease	Retro-Prospective	- Cross-comparison with a benchmark	15	The study will provide real-world clinical data essential for benchmarking and feedback activity, to positively impact outcomes (...)
Crozier-Shaw G, Hughes AJ, Cashman J, Synnott K. Instant messaging apps and data protection: combining to improve hip fracture care? Ir J Med Sci. 2022 Apr;191(2):765–9.	Trauma	- Prospective	- Cross-comparison with a benchmark	3	Streamlining communication through messaging services has and continues to be vital to improving care for hip fracture (...)
Opgenorth D, Reil E, Lau V, Fraser N, Zuege D, Wang X, et al. Improving the quality of the performance and delivery of continuous renal replacement therapy (CRRT) to critically ill patients across a healthcare system: QUALITY CRRT: a study protocol. BMJ Open. 2022 Feb 4;12(2):e054583.	Critical Care	Study Protocol	- Cross-comparison with a benchmark	10	QUALITY CRRT will test the application of this strategy stakeholder engagement and stepped-wedged implementation across an entire (...)

(*Continued*)

 © 2025. The Author(s).

Supplementary Appendix 1 (*Continued*)

Article	Declared Area of Interest / Science Related	Type of Experiment/ Study	KPIs Method	KPIs Quantity	Main Findings/ Results
Al Mutair A, Al Mutairi A, Schwebius D. The retention effect of staff education program: Sustaining a decrease in hospital-acquired pressure ulcers via culture of care integration. Int Wound J. 2021 Dec;18(6):843–9.	Intensive Care Unit	Retrospective	- Cross-comparison with a benchmark	1	Most importantly, after 3 years of implementation, the percentage (%) of HAPU among inpatients has further dropped.
Ratnovsky A, Rozenes S, Bloch E, Halpern P. Statistical learning methodologies and admission prediction in an emergency department. Australas Emerg Care. 2021 Dec;24(4):241–7.	Emergency Care	Retrospective	- Statistics	6	The selected indicators can be used to study whether emergency department allocates its resources properly to cope with overcrowding and the (…)
Crozier-Shaw G, Hughes AJ, Conlon B, Sheehan E, Merghani K. Hip fracture care during Covid-19: a regional trauma center's experience. Ir J Med Sci. 2021 Nov;190(4):1275–80.	Trauma and Orthopedic	Retrospective	- Cross-comparison with a benchmark	6	Despite improvements in hip fracture care KPIs, the Covid-19 crisis was associated with increased 30-day mortality in hip fracture patients.
Martin E, Beckmann M, Blythe R, Merollini K, Graves N. Adherence to best practice: Preventing surgical site infection following caesarean section in Australia. Aust N Z J Obstet Gynaecol. 2021 Oct;61(5):728–34.	Obstretician/ Gynecologist	Cross-Sectional	- Questionnary	Not listed	Adherence to best practice at caesarean section is low among many Australian obstetricians.
Carragher M, Steel G, O'Halloran R, Torabi T, Johnson H, Taylor NF, et al. Aphasia disrupts usual care: the stroke team's perceptions of delivering healthcare to patients with aphasia. Disabil Rehabil. 2021 Oct;43(21):3003–14.	Speech Pathology	Phenomenological Methodology	- Questionnary	12	Health professionals want to help but are working in a non-optimal environment where communication and patient-centered care are not(…)
Wang H, Kalloniatis M. Clinical outcomes of the Centre for Eye Health: an intra-professional optometry-led collaborative eye care clinic in Australia. Clin Exp Optom. 2021 Sep;104 (7):795–804.	Optometry	Retrospective	- Statistical	2	This integrated care pathway has the potential to reduce unnecessary referrals from optometrists to hospital ophthalmological service by offering(…)
Wu W, Shi L, Duan Y, Xu S, Shen L, Zhu T, et al. Nanobody modified high-performance AIE photosensitizer nanoparticles for precise photodynamic oral cancer therapy of patient-derived tumor xenograft. Biomaterials. 2021 Jul;274:120870.	Biomaterials	Design of Experiments	- Laboratory experimental	5	Under 532 nm clinical laser irradiation, the same mole of AIEPS5 could generate 3.78-fold more 1O2 than clinically approved Hemoporfin.

 © 2025. The Author(s).

Supplementary Appendix 1 (*Continued*)

Article	Declared Area of Interest / Science Related	Type of Experiment/ Study	KPIs Method	KPIs Quantity	Main Findings/ Results
Bormann CL, Curchoe CL, Thirumalaraju P, Kanakasabapathy MK, Gupta R, Pooniwala R, et al. Deep learning early warning system for embryo culture conditions and embryologist performance in the ART laboratory. J Assist Reprod Genet. 2021 Jul;38(7):1641–6.	Assisted Reproduction and Genetics	Exploratory	- Artificial intelligence<br>- Convolutional neural network	3	AI predictions in monitoring the performance of individual embryologist technical competency and early embryo developmental stage markers as a predictor for (...)
Chong SSF, Kim M, Limoli M, Obscherning E, Wu P, Feisee L, et al. Measuring Progress of Regulatory Convergence and Cooperation Among Asia-Pacific Economic Cooperation (APEC) Member Economies in the Context of the COVID-19 Pandemic. Ther Innov Regul Sci. 2021 Jul;55 (4):786–98.	Therapeutic Innovation	Exploratory	- Questionnary	8	Convergence efforts within APEC can accelerate availability of medical products including that related to COVID-19 vaccines, treatments and diagnostics (...)
Bhat S, Wells CI, Tan J, Bissett IP, Lill M. Colonoscopy quality indicators in patients with and without prior colonic resection: A single-center prospective comparative study. Colorectal Dis. 2021 Jul;23(7):1755–64.	Coloproctology	Prospective Cohort	- Statistical	3	Patients with nonintact colons comprise a small proportion of the overall colonoscopy cohort and it is unlikely that this small difference is relevant for (...)
Dietz HP. Ultrasound imaging of maternal birth trauma. Int Urogynecol J. 2021 Jul;32(7): 1953–62.	Urogynecology	Narrative Review	- Observational	Not listed	Translabial and exo-anal ultrasound allows the assessment of maternal birth trauma in routine clinical practice and the utilization of avulsion and sphincter trauma (...)
Network for Peri-operative Critical care (N4PCc)*. Addressing priorities for surgical research in Africa: implementation of a multicentre cloud-based peri-operative registry in Ethiopia. Anesthesia. 2021 Jul;76(7):933–9.	Anesthesia	Retrospective	- Statistical	11	This collaboration has successfully implemented a multicentre digital surgical registry that can enable measurement of key performance indicators for surgery and (...)
Tashkandi SA, Alenezi A, Bakhsh I, AlJuryyan A, AlShehry ZH, AlRashdi S, et al. Clinical laboratory services for primary healthcare centers in urban cities: a pilot ACO model of ten primary healthcare centers. BMC Fam Pract. 2021 May 27;22(1):105.	Pathology	Gap Analysis	- Statistical	3	Moreover, the model shed the light on how crucial the pre-analytical phase for laboratory quality improvement process, its effect on cost reduction (...)

(*Continued*)

Supplementary Appendix 1 (*Continued*)

Article	Declared Area of Interest / Science Related	Type of Experiment/ Study	KPIs Method	KPIs Quantity	Main Findings/ Results
Farag S, Feeney C, Lee V, Nagendran S, Jain R, Aziz A, et al. A “Real Life” Service Evaluation Model for Multidisciplinary Thyroid Eye Services. Front Endocrinol (Lausanne). 2021;12:669871.	Endocrinology	Retrospective Descriptive	- Statistical	7	This study forms a waymark for other units using TEAMeD-5 and BOPSS audit criteria.
Sreedher G, Ho ML, Smith M, Udayasankar UK, Risacher S, Rapalino O, et al. Magnetic resonance imaging quality control, quality assurance and quality improvement. Pediatr Radiol. 2021 May;51(5):698–708.	Radiology	Descriptive Research	- Authors background	6	Through the processes of quality control, quality assurance and quality improvement, clinical MR workflow is constantly being evaluated and improved for (...)
Al-Jazairi AS, Horanieh BK, Alswailem OA. The usefulness of an ambulatory care pharmacy performance dashboard during the COVID-19 pandemic in a complex tertiary care system. Am J Health Syst Pharm. 2021 Apr 22;78(9):813–7.	Pharmaceutical	Design of Experiments	- Statistics	4	Developing a dynamic, semi–real-time pharmacy dashboard during unstable circumstances such as those that have arisen during the COVID-19 pandemic can be very (...)
Impouma B, Wolfe CM, Mboussou F, Farham B, Saturday T, Pervilhac C, et al. Monitoring and evaluation of COVID-19 response in the WHO African region: challenges and lessons learned. Epidemiol Infect. 2021 Apr 14;149:e98	Epidemiology and Infection	Exploratory	- Web base database	10	Framework for COVID-19 built on the lessons learned from EVD and HIV, the development of an adaptable M&E framework (...)
Elsworthy N, R Blair M, Lastella M. On-field movements, heart rate responses and perceived exertion of lead referees in Rugby World Cup matches, 2019. J Sci Med Sport. 2021 Apr;24 (4):386–90.	Science and Medicine in Sport	Descriptive, Observational	- Data collection through attached devices	8	Referees covered on average 6674 ± 566 m (65.8 ± 6.3 m min−1), with 586 ± 290 m in high-speed running. Mean heart rate was 146 ± 9 beats min−1, summated-heart-rate- (...)
Argaw MD, Fekadu BD, Mamo E, Abebe MG, Rogers D, Demelash A, et al. Implementing a Social Accountability Approach for Maternal, Neonatal, and Child Health Service Performances in Ethiopia: A Pre-Post Study Design. Glob Health Sci Pract. 2021 Mar 31;9(1):123–35.	Primary Health Care	Longitudinal	- Statistics	11	The use of CSCs in Ethiopia contributed to the health system’s performance in terms of maternal and child health services.
Sánchez-Úbeda EF, Sánchez-Martín P, Torrego-Ellacuría M, Rey-Mejías ÁD, Morales-Contreras MF, Puerta JL. Flexibility and Bed Margins of the Community of Madrid’s Hospitals during the First Wave of the SARS-CoV-2 Pandemic. Int J	Public Health	Case Study	- Linear Hinges model	4	The public and private hospitals of the CoM were able to increase the number of available beds from 18,692 on 18 March 2020 to 23,623 on 2 April 2020.

 © 2025. The Author(s).

Supplementary Appendix 1 (*Continued*)

Article	Declared Area of Interest / Science Related	Type of Experiment/ Study	KPIs Method	KPIs Quantity	Main Findings/ Results
Environ Res Public Health. 2021 Mar 28;18 (7):3510					
Reichmuth D, Olstad BH, Born DP. Key Performance Indicators Related to Strength, Endurance, Flexibility, Anthropometrics, and Swimming Performance for Competitive Aquatic Lifesaving. Int J Environ Res Public Health. 2021 Mar 26;18(7):3454.	Public Health	Case Study	- Statistical	27	The present study showed that sprint swimming performance, upper body, and leg strength are particularly important for competitive lifesaving.
Herold M, Kempe M, Bauer P, Meyer T. Attacking Key Performance Indicators in Soccer: Current Practice and Perceptions from the Elite to Youth Academy Level. J Sports Sci Med. 2021 Mar;20 (1):158–69	Sports Science	Exploratory	- Questionnary	12	This study provides novel findings that demonstrate practitioners' perception and implementation of key performance indicators.
Wong C, Blum IR, Louca C, Sparrius M, Wanyonyi K. A retrospective clinical study on the survival of posterior composite restorations in a primary care dental outreach setting over 11years. J Dent. 2021 Mar;106:103586.	Dentistry	Retrospective Clinical	- Statistics	6	Within the limitations of this study, it is concluded that the survival rate of composite restorations at the UPDA is similar to other values amongst undergraduate dental (...)
Amos S, Wiggins JB, Shaw EK, Hannah WN. The Annual Institutional Review-Key Performance Measures and Processes. J Grad Med Educ. 2021 Feb;13(1):119–22.	Medical Education	Observation	- Questionnary	7	Our results show a wide range of institutional responses though consensus was achieved on 7 key performance measures.
Practice Committees of the American Society for Reproductive Medicine and Society of Reproductive Biologists and Technologists. Electronic address: jgoldstein@asrm.org. A review of best practices of rapid-cooling vitrification for oocytes and embryos: a committee opinion. Fertil Steril. 2021 Feb;115(2):305–10.	Reproductive Medicine	Explanatory Design	- Authors background	11	Selection of optimized protocols, along with operator training, will result in increases in efficiency, consistency reliability, and safety.
Fabozzi G, Albricci L, Cimadomo D, Amendola MG, Sanges F, Maggiulli R, et al. Blastulation rates of sibling oocytes in two IVF culture media: an evidence-based workflow to implement newly commercialized products. Reprod Biomed Online. 2021 Feb;42(2):311–22.	Reproductive Medicine	Design of Experiments	- Statistics	Not listed	Blastulation rate among cohorts of sibling oocytes cultured in the same incubator is a fast, reliable and comprehensive performance (...)

(*Continued*)

Supplementary Appendix 1 (*Continued*)

Article	Declared Area of Interest / Science Related	Type of Experiment/ Study	KPIs Method	KPIs Quantity	Main Findings/ Results
Peng Y, Du X, Li X, Ji J, Wu Y, Gao R, et al. Associations Between Education Level and In-hospital Treatment and Outcomes Among Acute Coronary Syndrome in China. Am J Med Sci. 2021 Feb;361(2):253–60.	Cardiology	Case Control	- Medical records<br>- Statistics	8	Less educated patients were at higher risk for adverse clinical events; however this was explained by diferences in baseline characteristics.
Ungureanu AN, Brustio PR, Lupo C. Technical and tactical effectiveness is related to time-motion performance in elite rugby. J Sports Med Phys Fitness. 2021 Feb;61(2):167–74.	Sports Science	Case Study	- Statistical	20	Technical and tactical performance affects running activities differently for forward and backs. During training sessions, coaches should stimulate forward to be (...)
Bocchi EA, Moreira HT, Nakamuta JS, Simões MV, CLIMB-HF Study Group, Casas A de AL, et al. Implications for Clinical Practice from a Multicenter Survey of Heart Failure Management Centers. Clinics (Sao Paulo). 2021;76: e1991.	Cardiology	Cross-Sectional	- Questionnary<br>- Statistics	6	HF-DMPs are highly heterogeneous. New strategies for HF care should consider the present study highlights and clinical decision-making processes to improve HF(...)
Peng Y, Du X, Li X, Ji J, Wu Y, Gao R, et al. Association of renal insufficiency with treatments and outcomes in patients with acute coronary syndrome in China. Int J Cardiol. 2021 Jan 15;323:7–12.	Cardiology	Design of Experiments	- Statistics	30	Among Chinese ACS patients, those with renal insufficiency have a lower percentage of adherence to guideline-recommended treatments and worse clinical prognosis.
Halling B, Bergman M, Wijk K. Sense of Coherence and Lean-based leadership and alterations in sick leave and productivity at a steel wire manufacturing unit. Work. 2021;68(4): 1211–20.	*Industrial Management and Mechanical Engineering,*	*Industrial Management*	*Industrial Management*	*Industrial Management*	*Industrial Management*
Thorakkattil SA, Nemr HS, Al-Ghamdi FH, Jabbour RJ, Al-Qaaneh AM. Structural and operational redesigning of patient-centered ambulatory care pharmacy services and its effectiveness during the COVID-19 pandemic. Res Social Adm Pharm. 2021 Jan;17(1): 1838–44.	Clinical Pharmacy	Retrospective<br>Comparative	- Statistics<br>- TI resources	7	As a result of the structural and operational changes made in patient-centered ambulatory care pharmacy services during the COVID-19 pandemic, a 48% prescriptions requests and 90% prescriptions fills are increased (...)

 © 2025. The Author(s).

Supplementary Appendix 1 (*Continued*)

Article	Declared Area of Interest / Science Related	Type of Experiment/ Study	KPIs Method	KPIs Quantity	Main Findings/ Results
Ramirez-Rodriguez JM, Martinez-Ubieto J, Muñoz-Rodes JL, Rodriguez-Fraile JR, Garcia-Erce JA, Blanco-Gonzalez J, et al. Surgical treatment for colorectal cancer: analysis of the influence of an enhanced recovery program on long-term oncological outcomes-a study protocol for a prospective, multicentre, observational cohort study. BMJ Open. 2020 Oct 27;10(10):e040316.	Oncology	Prospective Cohort	- Questionnary<br>- Statistics	18	Not listed
Chen Y, Zhong W, Gong X, Hu H, Yan S, Zhang X, et al. Rationale and design of individualized quality improvement based on the Computer Analyzing system to improve Stroke management quality Evaluation (CASE): a multicenter historically controlled study. Trials. 2020 Jul 24;21(1):677.	Stroke	Cohort	- Statistics<br>- TI resources	13	In summary, this is a new mode to improve in-patient medical care, which consists of advanced acquisition technique and personalized feedback with (...)
Wing CE, Turner AN, Bishop CJ. Importance of Strength and Power on Key Performance Indicators in Elite Youth Soccer. J Strength Cond Res. 2020 Jul;34(7):2006–14.	Science and Technology	Case Study	- Statistical	5	These data support the notion that strength and power training are important to soccer performance, particularly when (...)
Jones B, McClean S, Stanford D. Modelling mortality and discharge of hospitalized stroke patients using a phase-type recovery model. Health Care Manag Sci. 2019;22(4):570–88.	Health Care	Case Study	- Statistics	16	We observe that as patients age, more Haemorrhagic patients tend to die and fewer are discharged to their usual residence.
Zecchin R, Candelaria D, Ferry C, Ladak LA, McIvor D, Wilcox K, et al. Development of Quality Indicators for Cardiac Rehabilitation in Australia: A Modified Delphi Method and Pilot Test. Heart Lung Circ. 2019 Nov;28(11):1622–30.	Cardiovascular Health and Rehabilitation	Case Study	- Questionnary<br>- Statistics	12	The MDS of 11 QIs for CR provides an important foundation for collection of data to promote the quality of CR nationally and the opportunity to participate in (...)
Lima T de M, Aguiar PM, Storpirtis S. Development and validation of key performance indicators for medication management services provided for outpatients. Res Social Adm Pharm. 2019 Sep;15(9):1080–7.	Pharmacy	Case Study	- Questionnary<br>- Statistical	6	A set of 6 KPIs was developed for medication management services provided for outpatients.

(*Continued*)

Supplementary Appendix 1 (*Continued*)

Article	Declared Area of Interest / Science Related	Type of Experiment/ Study	KPIs Method	KPIs Quantity	Main Findings/ Results
Nag N, Tran L, Fotis K, Smith JA, Shardey GC, Baker RA, et al. Structured Feedback: Acceptability and Feasibility of a Strategy to Enhance the Role of a Clinical Quality Registry to Drive Change in Cardiac Surgical Practice. Heart, Lung and Circulation. 2019 Aug;28(8):1253–60.	Cardiac and Thoracic Surgeons	Cross-Sectional	- Questionnary<br>- Statistical	12	In this pilot study, compared with the control method, structured feedback did not significantly improve communication.
Wu Y, Li S, Patel A, Li X, Du X, Wu T, et al. Effect of a Quality of Care Improvement Initiative in Patients With Acute Coronary Syndrome in Resource-Constrained Hospitals in China: A Randomized Clinical Trial. JAMA Cardiol. 2019 May 1;4(5):418–27.	Cardiology	Cross-Sectional	- Data gathering<br>- Statistical	16	Among resource-constrained Chinese hospitals, introducing a multifaceted QCI intervention had no significant effect on in-hospital MACE, although it improved (...)
Woodward T, Hocking J, James L, Johnson D. Impact of an emergency department-run clinical decision unit on access block, ambulance ramping and National Emergency Access Target. Emerg Med Australas. 2019 Apr;31(2):200–4.	Emergency Medicine	Retrospective	- Data gathering<br>- Statistical	10	In summary, this ED led, consultant run CDU model of care resulted in significantly improved performance on a range of KPIs (...)
Dohmen PJG, van Raaij EM. A new approach to preferred provider selection in health care. Health Policy. 2019 Mar;123(3):300–5.	Health Policy	Retrospective	- Data gathering<br>- Statistical	10	This new approach focuses purely on provider selection and is thus complementary to innovations in health care reimbursement, such as value (...)
Siau K, Hodson J, Valori RM, Ward ST, Dunckley P. Performance indicators in colonoscopy after certification for independent practice: outcomes and predictors of competence. Gastrointest Endosc. 2019 Mar;89(3):482–492.e2.	Gastrointestinal Endoscopy	Prospective	- Data gathering<br>- Statistical	14	It is possible to identify predictors of underperformance in trainees, which may be of value to training leads and could improve the patient experience.
García-Alonso CR, Almeda N, Salinas-Pérez JA, Gutiérrez-Colosía MR, Uriarte-Uriarte JJ, Salvador-Carulla L. A decision support system for assessing management interventions in a mental health ecosystem: The case of Bizkaia (Basque Country, Spain). PLoS One. 2019;14(2):e0212179.	Mental Health	Design of Experiments	- TI resources<br>- Monte Carlo simulation	Not listed	Decision makers reported that the EDeS-MH is useful for helping them to (i) structure their minds in designing new interventions (...)
Nicholson A. Toward a more positive view of healthcare in Ireland. Ir J Med Sci. 2019 Feb;188(1):35–41.	Medical Science	Action Research	Not applicable	Not listed	Not applicable

 © 2025. The Author(s).

Supplementary Appendix 1 (*Continued*)

Article	Declared Area of Interest / Science Related	Type of Experiment/ Study	KPIs Method	KPIs Quantity	Main Findings/ Results
Spackman E, Clement F, Allan GM, Bell CM, Bjerre LM, Blackburn DF, et al. Developing key performance indicators for prescription medication systems. PLoS One. 2019;14(1): e0210794.	Community Health Sciences	Action Research	- RAND/UCLA	6	These indicators are recommended as a starting point to assess the current performance of prescription medication systems.
Kabbani S, Al Habeeb W, Liew HB, Mohan JC, Ogola E, Sim D, et al. Supporting the Management of Patients with Heart Failure within Asia-Pacific, Middle East, and African Countries: A Toolbox for Healthcare Providers. Cardiology. 2019;142 Suppl 1:1–10.	Cardiology	Action Research	Not applicable	Not listed	In this article, we present a HF Toolbox that provides practical resources to guide the management of patients with HF (AHF and chronic HFrEF) and (...)
Fitzpatrick PE, Greehy G, Mooney MT, Flanagan F, Larke A, Connors A, et al. Evolution of the National Breast Screening Program in Ireland: Two-year interval analysis (2004–2013) of BreastCheck. J Med Screen. 2018 Dec;25 (4):191–6.	Breast Cancer	Retrospective	- Data gathering<br>- Statistical	18 (Table 2)	Revised indicators to reflect the digital mammography era are anticipated in revised European Guidelines on breast cancer screening.
Philippon DJ, Marchildon GP, Ludlow K, Boyling C, Braithwaite J. The comparative performance of the Canadian and Australian health systems. Healthc Manage Forum. 2018 Nov;31(6):239–44.	Public Health	Retrospective	- Data gathering<br>- Statistical	35 (Table 3)	We conclude that while performance ratings of Australia are<br>generally better than Canada, the differences are a matter of degree.
Liu J, Schatzkin E, Omoluabi E, Fajemisin M, Onuoha C, Erinfolami T, et al. Introducing the subcutaneous depot medroxyprogesterone acetate injectable contraceptive via social marketing: lessons learned from Nigeria's private sector. Contraception. 2018 Nov;98 (5):438–48.	Contraception	Cross-Sectional	- Interview<br>- Phone survey<br>- Interview	Not informed	In the DMPA-SC introductory program in Nigeria, distribution was amplified when focused on high-volume contraceptive providers.
Obaro AE, Burling DN, Plumb AA. Colon cancer screening with CT colonography: logistics, cost-effectiveness, efficiency and progress. Br J Radiol. 2018 Oct;91(1090):20180307.	Radiology	Action Research	- Data gathering	Not listed	Not applicable
Penverne Y, Leclere B, Labady J, Berthier F, Jenvrin J, Javaudin F, et al. Key performance indicators' assessment to develop best practices in an Emergency Medical Communication Centre. Eur J Emerg Med. 2018 Oct;25(5):335–40.	Emergency Medicine	Prospective	- Database<br>- Statistics	4	The occupation rate appeared to be the most important factor contributing toward the QS20. Our data will be useful to develop (...)

 © 2025. The Author(s).
